# Analysis of the Effects of Sugar Modifications on RNA Chemical Ligation Reactions

**DOI:** 10.1002/cbic.202500263

**Published:** 2025-10-28

**Authors:** Yu Hirano, Naoshi Kojima, Harei Sakurai, Emi Saito, Hirokazu Nankai, Yasuo Komatsu

**Affiliations:** ^1^ Biomanufacturing Process Research Center National Institute of Advanced Industrial Science and Technology (AIST) 2‐17‐2‐1 Tsukisamu‐Higashi Sapporo 062‐8517 Japan; ^2^ Health and Medical Research Institute National Institute of Advanced Industrial Science and Technology (AIST) AIST Tsukuba Central 6 1‐1‐1 Higashi Tsukuba Ibaraki 305‐8566 Japan; ^3^ GeneDesign Inc. dba Ajinomoto Bio‐pharma Services 7‐7‐29 Saitoasagi Ibaraki Osaka 567‐0085 Japan; ^4^ Cellular and Molecular Biotechnology Research Institute National Institute of Advanced Industrial Science and Technology (AIST) AIST Tsukuba Central 6 1-1-1 Higashi, Tsukuba Ibaraki 305‐8566 Japan

**Keywords:** chemical ligation, microRNA, modified nucleotide, nucleic acid, RNA

## Abstract

Chemical ligation of RNA fragments is an effective method for synthesizing long RNAs. It is particularly useful for incorporating site‐specific modifications into long RNAs; however, its low reaction efficiency remains a major challenge. Herein, 5′‐amino‐2′‐substituted uridine or cytidine nucleotides are synthesized, which are attached to the 5′ end of a synthetic RNA and conjugated with a phosphate group at the 3′ end of an alternative RNA fragment, to perform a head‐to‐tail‐type RNA ligation. It is examined how the 2′ position of nucleotides with 5′‐amino or 3′‐phosphate groups in the ligated site affects the ligation reaction. The 2′‐fluoro‐5′‐amino‐nucleotide shows enhanced reactivity compared with the 2′‐ribo‐ or 2′‐deoxy‐5′‐amino‐nucleotide. In contrast, the 2′‐*O*‐methyl modification demonstrates optimal efficacy with the 3'‐phosphate nucleotide. Interestingly, the 2′‐fluoro‐5′‐amino nucleotide maintains remarkable reactivity, even under acidic conditions. A pre‐miRNA comprising 84 nucleotides is synthesized through ligation, and the intracellular functionality of the ligated RNA is confirmed. This study elucidates the effect of 2′ substituents on chemical ligation reactions, providing insights into the chemical synthesis of long RNAs.

## Graphical abstract

The effect of substitutions at the 2′ position of the nucleotides at the ligation site on phosphoramidate‐type chemical ligation reaction efficiency is examined. The efficiency varies depending on the substituents, with the combination of a fluorine group on 5′NRNA and an *O*‐methyl group on RNA3′P showing the highest efficiency.

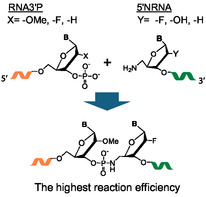

## 1 Introduction

RNA molecules with site‐specific chemical modifications are essential tools for examining RNA function in living cells.^[^ ^1^ ^]^ Although RNA strands may be prepared from DNA templates via in vitro transcription, this method only allows for the incorporation of functional groups at random positions. In contrast, chemical synthesis performed on a solid support enables the synthesis of short RNA with specific site modifications. Long RNAs with site‐specific chemical modifications are accessible through enzymatic or chemical ligation of short RNA strands; however, assembly or synthesis of long‐modified RNAs remains challenging.^[^ ^2^, ^3^ ^]^

Chemical synthesis methods for RNA can be readily scaled up and are more cost‐effective than enzymatic methods. Chemical ligation enables the formation of covalent bonds between two oligonucleotides (ONs) in the presence of a template ON.^[^ ^4^, ^5^ ^]^ However, chemical ligation between RNA fragments to form a native phosphodiester linkage often results in low yields.^[^ ^4^ ^]^ Thus, phosphoramidate‐type ligation reactions have been developed for RNA synthesis. Using 1‐ethyl‐3‐(3‐dimethylaminopropyl) carbodiimide (EDC), a primary amine at the 3′ end of RNA can be linked to a phosphate group at the 5′ end to produce RNA with a phosphoramidate structure.^[^ ^4^, ^6^, ^7^ ^]^ In addition, a primary amine at the 5′ end of RNA can be linked to 3′‐phosphorothioate‐RNA with 1‐fluoro‐2,4‐dinitrobenzene (DNFB).^[^ ^8^ ^]^ Although phosphoramidate RNA ligation reactions exhibit a higher reaction efficiency than phosphodiester ligations, the reaction efficiency is ≈50% for the EDC‐mediated reaction and 80% for the DNFB‐mediated reaction. Consequently, chemical ligation reactions for RNA require a significant amount of time, making sequential reaction of multiple ONs during the synthesis of long RNA molecules challenging.

**Figure 1:**
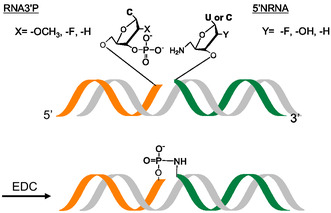
Chemical ligation reaction between 2′‐X modified 3′‐phosphorylated RNA (RNA3′P) and 2′‐Y modified 5′‐amino (5′NRNA).

Several ON 2′ modifications have been introduced to enhance duplex stability and increase resistance to enzymatic hydrolysis.^[^ ^1^, ^9^, ^10^ ^]^ We hypothesized that 2′ modifications contribute to the reactivity of primary amine and phosphate groups at the ligation site to improve the reaction efficiency of phosphoramidate‐type ligation. In this study, a phosphoramidite unit was synthesized to generate 5′‐amino RNA with a 2′ modification at the 5′ end (5′NRNA). In addition, RNA with a phosphate group at the 3′ end (RNA3′P) was synthesized with various 2′ modifications. We evaluated the ligation reaction between RNA3′P and 5′NRNA (**Figure** **1**). The efficiency of RNA ligation varied with 2′ modification. The optimized protocol yielded over 90% conversion within 30 min for the combination of 5′NRNA with a 2′‐fluorine modification and RNA3′P with a 2′‐Ome modification. Finally, we have herein described the synthesis and evaluation of pre‐miRNA prepared using this chemical ligation method.

## 2 Results and Discussion

### 2.1 Preparation of ONs with 5′‐Amino or 3′‐Phosphate Groups

**Figure 2:**
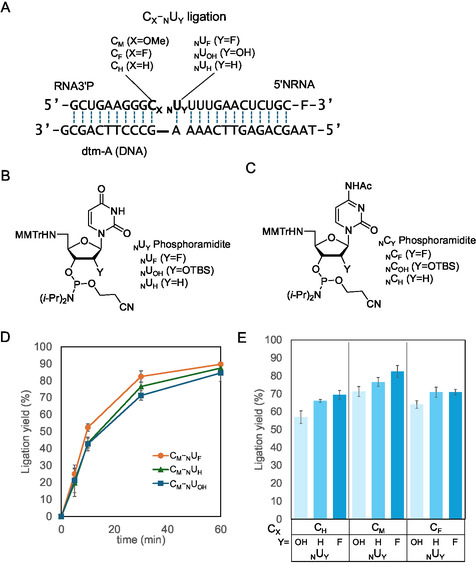
Chemical ligation of 3′‐phosphorylated RNA (RNA3′P =C_X_) and 5'‐amino modified RNA (5′NRNA = _N_U_Y_) on template DNA (dtm‐A). A) Sequence of the strands and their 2′‐modification at the conjugation site. Structure of B) uridine and C) cytosine phosphoramidite reagents used for the synthesis of 5′‐amino‐modified oligonucleotides. D) Time course of the ligation yield (C_M_ and _N_U_Y_) was determined based on the fluorescence intensity of the product (mean ± SD, *n *= 3–5). E) Comparison of ligation yields (C_X_ and _N_U_Y_) from a 30 min reaction. A reaction solution containing 0.1 μM RNA3′P, 0.1 μM 5′NRNA, and 0.1 μM dtm‐A was incubated in 50 mM HEPES buffer (pH 7.2) containing 100 mM NaCl at 37 °C. (mean ± SD, *n* = 3–5).

We performed a phosphoramidate‐type RNA ligation of 10 mer (RNA3′P) and 13 mer (5′NRNA) RNAs hybridized with a DNA template (dtm‐A) (**Figure** **2**A). To introduce an amino group at the 5′ end, 5′‐amino‐3′‐amidite uridine and cytidine analogs (_N_U_Y_ and _N_C_Y_, respectively) were first synthesized (Figure 2B,C). The 2′‐position atom (Y) was substituted with fluorine (F), hydroxy (OH), or hydrogen (H) to determine whether the 2′ modification could affect the ligation reaction. The 2′ position (X) of the 3′‐terminal cytidine (C_x_) with a phosphate group was also substituted with F, 2′‐*O*‐methyl (OMe), or H. To avoid 2′,3′‐self cyclization in the presence of a condensing agent, the 2′‐hydroxyl group was not used for X.

### 2.2 Analysis of the Effect of 2′ Modification on Phosphoramidate‐Type Ligation Reaction

We first performed conjugation between C_X_ and _N_U_Y_ (C_X_‐_N_U_Y_) in the presence of the condensing agent EDC after hybridization with a DNA template (Figure 2A) at 37 °C. For all combinations of C_X_‐_N_U_Y_, the ligation products were detected within 5 min after initiating the reaction (Figure S2, Supporting Information), and the molecular weights were confirmed (Table S1, Supporting Information). Although EDC can potentially react with uracil and guanine residues during RNA structure probing,^[^ ^11^, ^12^ ^]^ no detectable byproducts were observed in the high performance liquid chromatography (HPLC) profiles (Figure S3, Supporting Information), indicating that such side reactions were negligible under our conditions. As shown in Figure 2D, _N_U_F_ in 5′NRNA exhibited higher reactivity to C_M_ in RNA3′P than _N_U_H_ and _N_U_OH_. The same trend was observed for C_F_ and C_H_ in RNA3′P (Figure 2E). C_M_ produced more ligation products than C_H_ and C_F_ (Figure 2E). Overall, C_M_‐_N_U_F_ was the best combination for producing ligated products. The effect of 2′ modifications at the conjugation sites (C_X‐N_U_Y_) was also evaluated using an RNA template. Similar to the template DNA, the combination of C_M_ and _N_U_F_ exhibited the highest yield (Figure S4, Supporting Information). These results indicated that the 2′ modification at the conjugation site significantly influenced the reaction efficiency.

**Figure 3:**
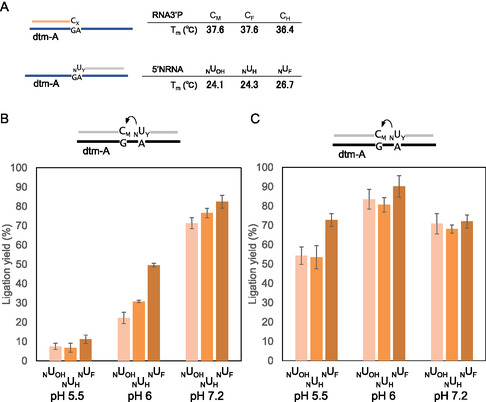
Chemical ligation reaction at different pH values. A) T_m_ values in hybridization between dtm‐A and 5′NRNA or RNA3′P. Comparison of the ligation yields of C_M_‐_N_U_Y_ for the 30 min reaction B) at 37 °C and C) 27 °C. A solution containing 0.1 μM RNA3′P, 0.1 μM 5′NRNA, and 0.1 μM template DNA was incubated in 50 mM MES (pH 5.5 and 6) or HEPES buffer (pH 7.2) containing 100 mM NaCl (mean ± SD, *n *= 3–5).

Thermal denaturation experiments were conducted to evaluate the hybridization stability of RNA3′P or 5′NRNA with the DNA template (dtm‐A). RNA3′P with C_M_, C_F_, or C_H_ at the 3′ end showed nearly identical T_m_ values of ≈37 °C (**Figure** **3**A, C_M_ 37.6 °C, C_F_ 37.6 °C, or C_H_ 36.4 °C). In contrast, 5′NRNA exhibited a T_m_ ≈ 10 °C lower than that of RNA3′P because of a lower GC content. The hybridization stability of 5′NRNA significantly affected the ligation efficiency compared with RNA3′P. The T_m_ value of the _N_U_F_ complex was slightly higher than that of the _N_U_OH_ and _N_U_H_ complexes (Figure 3A). Notably, 5′NRNA with _N_U_F_ exhibited a higher ligation yield at 37 °C (Figure 2E). This may be the result of the stabilizing effect of the 2′‐fluoro modification.^[^ ^13^ ^]^

The carbodiimide‐mediating reaction is generally accelerated by acidic conditions at around pH 4.5;^[^ ^14^ ^]^ however, the reaction rate between the phosphate and amino groups is reduced at lower pH values because of amino group protonation.^[^ ^8^ ^]^ Next, we performed a C_M_‐_N_U_Y_ ligation (Y = OH, H, or F) in a different pH solution. The percentage of ligation products after 30 min at 37 °C is shown in Figure 3B. At pH 7.2, the reaction products were obtained at ≈70%–80% yield, and _N_U_F_ exhibited higher reactivity than _N_U_H_ and _N_U_OH_. At pH 6.0, the ligation yields of _N_U_OH_ and _N_U_H_ significantly decreased to ≈20%–30%, whereas _N_U_F_ maintained a higher efficiency of ≈50%. Under more acidic conditions (pH 5.5), the ligation yields decreased further to ≈ 10%, with _N_U_F_ displaying 1.5‐fold higher ligation yields than _N_U_OH_ and _N_U_H_. Thus, under all pH conditions, the ligation yield of _N_U_F_ was higher than that of _N_U_H_ and _N_U_OH_, and this difference became more pronounced at pH 6.0. These results indicated that _N_U_F_ was more effective in the ligation reaction under mild acidic conditions. As mentioned above, the duplex‐stabilizing effect of the 2′‐fluoro modification may have increased the ligation ratio at 37 °C. To minimize the duplex‐stabilizing effect of the 2′‐modification on the ligation reaction, we performed the reaction at a lower temperature (27 °C). At pH 7.2, C_M‐N_U_OH_, C_M‐N_U_H_, and C_M‐N_U_F_ exhibited similar ligation yields at 27 °C (Figure 3C). The results indicated that 2′‐modifications had little impact on the ligation yields under neutral conditions and at 27 °C, where the duplex complex was stable. However, at pH 6.0, the ligation yields increased by ≈10% for all RNA3′Ps, and _N_U_F_ exhibited the highest reactivity, reaching 90%, compared with _N_U_H_ and _N_U_OH_. Under more acidic conditions (pH 5.5), the ligation yields of _N_U_OH_ and _N_U_H_ decreased to ≈50%, whereas _N_U_F_ still exhibited a higher efficiency of ≈ 70% (Figure 3B).

Next, we conducted ligation reactions using 5′‐amino‐modified cytidine (_N_C_Y_; Y = OH, H, or F; Figure S5A, Supporting Information) in the presence of a template DNA (dtm‐G; Figure S5A, Supporting Information) that was complementary to 5′NRNA under varying pH conditions. The percentages of the ligation products after 30 min at 37 °C are shown in Figure S5B, Supporting Information. At pH 7.2, _N_C_Y_ exhibited a reaction profile almost identical to that of _N_U_Y_. In contrast, at pH 6.0, the ligation yield of C_M_‐_N_C_F_ increased by ≈10%, and _N_C_F_ showed higher reactivity than _N_C_H_ and _N_C_OH_ (Figure S5B, Supporting Information). The increase in the ligation yield of _N_C_F_ was similar to that of _N_U_F_ at 27 °C and was thought to be influenced by the higher T_m_ resulting from the increased GC content. Under more acidic conditions (pH 5.5), the ligation yields of _N_C_OH_ and _N_C_H_ decreased to ≈ 20%–30%, whereas _N_C_F_ still exhibited a higher efficiency of ≈50% (Figure S5B, Supporting Information). At 27 °C, the ligation yield of C_M_‐_N_C_F_ increased under mild acidic conditions (pH 6.0) and was higher than that of _N_C_OH_ and _N_C_H_. This indicated that _N_C_Y_ exhibits a reaction profile similar to _N_U_Y_ (Figure 3B,C).

Notably, the ligation yields of _N_U_OH_ and _N_U_H_ (or _N_C_OH_ and _N_C_H_) were suppressed more at pH 5.5 than that of _N_U_F_ (or _N_C_F_) (Figure 3B,C, and S4B, Supporting Information, C). These results suggest that the protonation of the 5′‐amino group in _N_U_F_ (or _N_C_F_) is suppressed because of the electron‐withdrawing effect of the 2′‐fluorine modification, which lowers the pKa. In other words, the 2′‐fluorine modification enables the efficient ligation of carbodiimide, even under acidic conditions.

### 2.3 Synthesis of pre‐miRNA with Phosphoramidate‐Type Ligation

**Figure 4:**
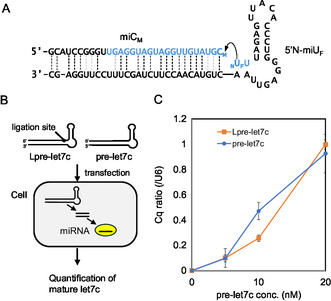
Synthesis of pre‐miRNA (84 mer) through chemical ligation between 2′‐Ome‐3′‐phosphate‐RNA (miC_M_) and 5′‐amino‐2′‐fruoro‐RNA (5′N‐miU_F_). The blue text indicates the sequence of let‐7c. A) Sequencing of each oligonucleotide and its 2′ modification of conjugation site. B) Schematic representation of the introduction of pre‐let7c into cells and quantification of mature let7c. C) qPCR analysis of let7c in HepG2 cells 48 h after transfection with pre‐miRNA. (mean ± SD, *n *= 3).

We synthesized pre‐let‐7c (84 mer) from 54 mer (5'N‐miU_F_) and 30 mer (miC_M_) RNAs via phosphoramidate‐type ligation (**Figure** **4**A). Because 5′N‐miU_F_ with 5′‐amino‐2′‐fluoro‐uridine consists of a complementary sequence to miC_M_ that contains 2′‐OMe‐3′‐phosphate modifications at the 3′ end, this ligation was performed without a template strand. As a result, the ligated product was efficiently produced, and the starting RNA fragments nearly disappeared after 120 min, whereas the conversion yields exceeded 90% (Figure S6, Supporting Information). The main product was confirmed to be the full‐length pre‐let‐7c containing a phosphoramidate linkage at the ligation site (Lpre‐let‐7c, MW: calcd. 26984.13, found 26984.3658). Thus, the use of a phosphoramidate‐type ligation between 2′‐OMe‐3′‐phosphate RNA and 5′‐amino‐2′‐fluoro‐RNA facilitated the rapid and efficient synthesis of pre‐miRNA. For comparison, we also prepared pre‐let‐7c as a control through solid‐phase chemical synthesis. When pre‐miRNA is transfected into cells, it is processed by Dicer and loaded into the Argonaute protein, resulting in the generation of mature miRNA (Figure 4B).^[^ ^15^, ^16^ ^]^ We transfected Lpre‐let‐7c or pre‐let‐7c into cells and quantified the mature miRNA levels to determine whether these synthetic pre‐miRNAs could be converted into mature miRNA. The miRNA levels in the cells increased proportionally with the pre‐miRNA concentration applied to the cell culture media (Figure 4C). These results suggest that long‐chain RNAs capable of functioning within cells can be synthesized using a phosphoramidate‐type ligation.

We hypothesized that 2′ modifications enhance the reactivity of both the amino and phosphate groups at the ligation site to improve the efficiency of the phosphoramidate‐type ligation. In this study, we prepared two RNA fragments containing various 2′ modifications at the terminal nucleotides. The efficiency of RNA ligation was dependent on the type of 2′ modification.

2′‐Fluoro modification at the sugar moiety of RNA stabilizes the duplex by locking the sugar conformation predominantly in the C3′‐*endo* conformation.^[^ ^17^ ^]^ In addition, the electron‐withdrawing effect of fluorine propagates through the entire nucleobase, shortening the distance between bases.^[^ ^18^ ^]^ Because the fluorine atom is small and does not interfere with RNA binding proteins, 2′‐fluoro‐modified RNAs are widely used as biological probes such as short interfering RNA and miRNAs.^[^ ^13^, ^17^ ^]^ In this study, we used the 2′‐fluoro modification in the RNA chemical ligation reaction to synthesize long RNA molecules. To date, the effect of substitutions at the 2′‐position of RNA in chemical ligation reactions has not been examined.

The ligation reactions between 2′‐fluoro‐modified 5′NRNA (_N_U_F_) and 2′‐OMe‐modified 3′‐phosphate RNA (C_M_) exhibited a higher efficiency than those between other combinations of 2′ modifications (Figure 2E). The thermal denaturation experiment of the RNA fragment used for the ligation reaction revealed that the 2′‐fluoro modification stabilized the duplex formed with the DNA template. These results suggested that the stabilizing effect of the 2′‐fluoro modification on duplex formation at the ligation site may contribute to a higher efficiency ligation reaction. However, the effect of 2′‐OMe modification on RNA3′P was unclear as thermal denaturation studies did not show a preference for 2′ modifications. Furthermore, the ligation efficiency of 2′‐fluoro‐modified 5′NRNA was higher under acidic conditions than that of RNAs with other 2′‐modifications (Figure 3B), suggesting that the high electronegativity of the fluorine atom may have partially suppressed the protonation of the 5′‐amino group. Overall, these data indicate that the duplex‐stabilizing effect and strong electronegativity of the 2′‐fluoro modification contribute to its high ligation efficiency. Interestingly, at 37 °C, the ligation yield of C_M‐N_U_F_ at pH 6.0 decreased compared with that at pH 7.2, whereas at 27 °C, the ligation yield of C_M‐N_U_F_ was the highest at pH 6.0 (Figure 3B,C). This phenomenon may be the result of the destabilization of hybridization between RNA3′P and the template strand caused by reaction with EDC at 37 °C, thereby leading to a lower ligation yield under acidic conditions (pH 6.0). In contrast, the destabilizing effect of EDC on hybridization was minimized at 27 °C, during which the duplex was more stable, and the increased reactivity at EDC under acidic conditions resulted in a higher ligation yield.

The phosphoramidate‐type ligation reaction was applied to synthesize pre‐miRNA. We synthesized pre‐let7c containing 84 nucleotides from 2′‐fluorine and 2′‐*O*‐methyl terminal nucleosides via ligation and confirmed the functioning of the ligated RNA within cells. Because Lpre‐let‐7c and pre‐let‐7c showed almost identical results (Figure 4C), we concluded that a phosphoramidate‐type ligation could be used to synthesize long‐chain RNA capable of functioning within cells.

In conclusion, our results revealed that appropriate 2′‐modification of both 5′‐ and 3′‐ terminal nucleotides increases the reactivity of phosphoramidate‐type ligation reactions. Under mild acidic conditions, a high reaction efficiency of over 90% was achieved within 30 min. This suggests that long‐chain RNA with site‐specific chemical modifications can be synthesized at a low cost and without the need for enzymes. This simple and efficient technique holds promise for the synthesis of mRNA and noncoding RNA.

## 3 Experimental Section

3.1

3.1.1

##### Synthesis of Phosphoramidite Units

The synthesis of phosphoramidite monomers used to prepare 5′‐amino‐modified ONs^[^ ^19^, ^20^ ^]^ (Figure 2B,C) is detailed in the Supporting Information.

##### Preparation of ONs

All ONs were chemically synthesized using DNA/RNA phosphoramidite, solid supports, and reagents on an nS‐8 II automated DNA/RNA synthesizer (GeneDesign, Inc) using a standard 0.2 μmol or 1.0 μmol phosphoramidite cycle of detritylation, coupling, capping, and oxidation. RNAs were synthesized and deprotected using the standard TBDMS chemistry protocol provided by the manufacturer. Deprotected ONs were purified via reverse‐phase HPLC using an X‐Bridge C18 column with a gradient system of methanol in 100 mM hexafluoro‐2‐propanol (HFIP) and 8 mM triethylamine (TEA) on Shimadzu LC‐20 prominence. All ONs were characterized through electrospray ionization mass spectrometry (ESI‐MS, Xevo G2‐XS QToF, Waters).

##### T_m_ Measurements

Thermal denaturation experiments were performed for all duplexes (1.5 μM) in 10 mM sodium cacodylate buffer (pH 7) containing 10 mM NaCl. The solutions were incubated at 90 °C for 3 min and gradually cooled to anneal each duplex. The UV absorbance of the duplexes was measured using a UV‐2500PC (Shimadzu). The absorbance of the sample was measured at 260 nm between 5 °C and 90 °C using a ramp rate of 0.5 °C/min. The cuvette and Peltier block were equilibrated at 5 °C for 10 min prior to data acquisition. The absorbance of DNA/RNA or RNA/RNA was recorded at temperature intervals of 0.2 °C with a slit width of 1 nm. T_m_ was determined using the first derivative method and calculated using the T_m_ analysis software (Shimadzu).

##### Chemical Ligation Reactions

Ligation reactions were performed in a 20‐μL volume containing 0.1 μM RNA3′P, 0.1 μM 5′NRNA, and 0.1 μM template ON in 50 mM HEPES buffer (pH 7.2) or 50 mM MES buffer (pH 5.5 or 6.0), both containing 100 mM NaCl. The ON solutions were annealed by heating at 90 °C for 5 min, followed by slow cooling to room temperature. EDC·HCl (final concentration: 600 mM) was added to the annealed ONs, and the reaction mixture was incubated at 17 °C, 27 °C, 37 °C, or 42 °C. At appropriate time points, 3 μL aliquots were mixed with 12 μL of loading buffer (80% formamide, 10 mM EDTA). Reactions were analyzed via electrophoresis on a 15% denaturing polyacrylamide gel (PAGE; 5.6 M urea, 25% formamide, 1× TBE) and visualized using Typhoon FLA 9000 (GE Healthcare Life Sciences). The chemical ligation yield was determined by comparing the relative fluorescence intensities of 5′NRNA and the ligated product.

To confirm the molecular weight of the ligation products, large‐scale reactions (500 μL) were performed using 1 μM each of RNA3′P, 5′NRNA, and the template ON (Figure S3, Supporting Information). The ON solutions were annealed by heating at 90 °C for 5 min, followed by slow cooling to room temperature. EDC·HCl (final concentration: 600 mM) was added to the annealed ONs, and the reaction mixture was incubated at 37 °C. The ligated products were desalted using a Sephadex NAP‐5 column and purified via reverse‐phase HPLC on a μ‐Bondasphere C18 column (Waters) with a gradient of acetonitrile and 0.1 M triethylammonium acetate (TEAA) buffer using a Gilson HPLC system. The products were characterized using electrospray ionization mass spectrometry (Q Exactive Plus, Thermo Fisher Scientific).

##### Synthesis of pre‐Let7c

To synthesize pre‐let7c, a ligation reaction was performed between a 30‐mer RNA fragment with a phosphate group and 2′‐*O*‐methyl at the 3′ end (miC_M_) and an RNA fragment with an amino group and 2′‐fluorine at the 5′ end (5′N‐miU_F_). A solution containing 2 μM miCM and 2 μM 5′N‐miUF was incubated at 27 °C in 50 mM HEPES buffer (pH 7.2) containing 100 mM NaCl. The ON solutions were annealed by heating at 90 °C for 5 min, followed by slow cooling to room temperature. EDC·HCl (final concentration: 600 mM) was added to the annealed ONs, and the reaction mixture was further incubated at 27 °C for 120 min. The ligated products (Lpre‐let7c) were processed as described above, including desalting with a Sephadex NAP‐5 column, purification via reverse‐phase HPLC, and characterization via Q Exactive Plus. For this ligation reaction, 5′N‐miUF acted as a template strand. A native pre‐miRNA molecule (pre‐let7c) was also prepared using a DNA/RNA synthesizer for use as a control.

##### Transfection of pre‐Let7c

To determine whether the ligated RNA molecule was functional, synthesized pre‐let7c was transfected into cells, and the amount of mature let7c was quantified using quantitative polymerase chain reaction (qPCR). Single‐stranded miRNAs of 17–24 nucleotides are small regulatory RNAs expressed in animals and plants. They can affect the translation or stability of target mRNAs.^[^ ^21^, ^22^, ^23^ ^]^ In the nucleus, pre‐miRNAs are cleaved from primary transcripts called “pri‐miRNAs” by Drosha. Subsequently, pre‐miRNAs are transported to the cytoplasm, where they are further cleaved by Dicer to generate short, double‐stranded RNAs, of which one strand becomes the mature miRNA.^[^ ^15^ ^]^ Therefore, the functionality of pre‐miRNA in living cells may be assessed using miRNA‐specific qPCR.^[^ ^24^ ^]^ The HepG2 cell line (hepatocellular carcinoma, RRID: CVCL_0027) was obtained from the RIKEN Cell Bank (RCB1648). HepG2 cells were cultured in Dulbecco's Modified Essential Medium (DMEM, Sigma) supplemented with 10% fetal bovine serum (FBS, Gibco), 100 units/ml penicillin (Gibco), and 100 μg ml^−1^ streptomycin (Gibco). The cells were seeded at a density of 2 × 10^4^ cells per well in 4‐well plates (Nunc) in DMEM (1 mL) containing 10% FBS. The following day, after removing an aliquot (500 μL) from each well, the cells were transfected in triplicate with DMEM (50 μL) containing Lpre‐let7c or pre‐let7c with Lipofectamine 2000 (Invitrogen; 1.5 μL per well). The concentration of Lpre‐let7c or pre‐let7c varied from 0 to 20 nM. After a 48‐h incubation, the cells were harvested using TRI Reagent (Molecular Research Center, USA). The miRNAs were isolated using the miRNeasy Mini Kit (Qiagen) according to the manufacturer's instructions. Complementary DNA (cDNA) was synthesized from 10 ng of miRNA using the TaqMan MicroRNA Reverse Transcription Kit (Thermo Fisher Scientific) according to the manufacturer's instructions. Real‐time polymerase chain reaction was performed using the TaqMan Universal Master Mix II with the LightCycler 96 system (Roche Diagnostics, Germany). U6 RNA was used as an endogenous control for normalizing let‐7c relative quantitation.

## Conflict of Interest

Harei Sakurai, Emi Saito, and Hirokazu Nankai are employees of GeneDesign, Inc. dba Ajinomoto Bio‐pharma Services.

## Supporting information

Supplementary Material

## Data Availability

The data that support the findings of this study are available in the supplementary material of this article.
