# Supplementary material for: Analysis of the Effects of Sugar Modifications on RNA Chemical Ligation Reactions
Source: Chembiochem. 2025 Oct 28;26(20):e202500263. doi: 10.1002/cbic.202500263 (PMC12582156; doi:10.1002/cbic.202500263)
Supplement: Supplementary file 1 — Supplementary Material [file CBIC-26-e202500263-s001.pdf]

Supporting Information

Analysis of the effects of sugar modifications on RNA chemical ligation reactions

Contents

1. Synthesis of phosphoramidite monomers of 5'-amino modified nucleosides.
2. Molecular weights of ligation products by phosphoramaidate-type ligation reaction (Table S1).
3. Chemical ligation of RNA with a phosphate group at the 3' end (RNA3'P: C_X) and RNA with an amino group at the 5' end (5N'RNA: $_NU_Y$) on template DNA (dtm-A).
4. Chemical ligation of C_X and NU_Y on template RNA (rtm-A).
5. Chemical ligation of 3'-phosphorylated RNA (RNA3'P: C_X) and 5'-amino modified RNA (5'NRNA: $_NC_Y$) on template DNA (dtm-G).
6. HPLC analysis of the ligation reaction between miCM and 5'N-miUF.

1. Synthesis of phosphoramidite monomers of 5'-amino modified nucleosides.

The phosphoramidite monomer of 5'-amino-2'-fluoro-2',5'-dideoxuuridine (${}_{N}U_{F}$, Fig. 2B) was synthesized as previously described with some modifications (Ref. 19 in main text). The phosphoramidite monomers of 5'-amino-2'-fluoro-2',5'-dideoxycytidine (${}_{N}C_{F}$), 5'-amino-5'-deoxyuridine (${}_{N}U_{OH}$), 5'-amino-5'-deoxycytidine (${}_{N}C_{OH}$), 5'-amino-2',5'-dideoxyuridine (${}_{N}U_{H}$), and 5'-amino-2',5'-dideoxycytidine (${}_{N}C_{H}$) were synthesized according to the method reported in Ref. 19 and Ref. 20 in main text.

General Information

Physical data were measured as follows: ^{1}H (500 MHz), ^{13}C (125 MHz), and ^{31}P (202 MHz) nuclear magnetic resonance (NMR) spectra were recorded on a Bruker Avance 500 NMR spectrometer with DMSO-d_6 as the solvent and tetramethylsilane (^{1}H and ^{13}C) or 85% phosphoric acid (^{31}P) as internal standards. Chemical shifts are reported in parts per million (δ), and signals are expressed as s (singlet), d (doublet), t (triplet), m (multiplet), or br (broad). Mass spectra were recorded on a Thermo Scientific Exactive at the Global Facility Center, Hokkaido University. Analytical thin layer chromatography (TLC) was performed on a Silicagel 70 TLC Plate (Fujifilm Wako Pure Chemical Corporation). The silica gel used for column chromatography was Nacalai Silica Gel 60 (particle size 150 μm) (Nacalai Tesque, INC.).

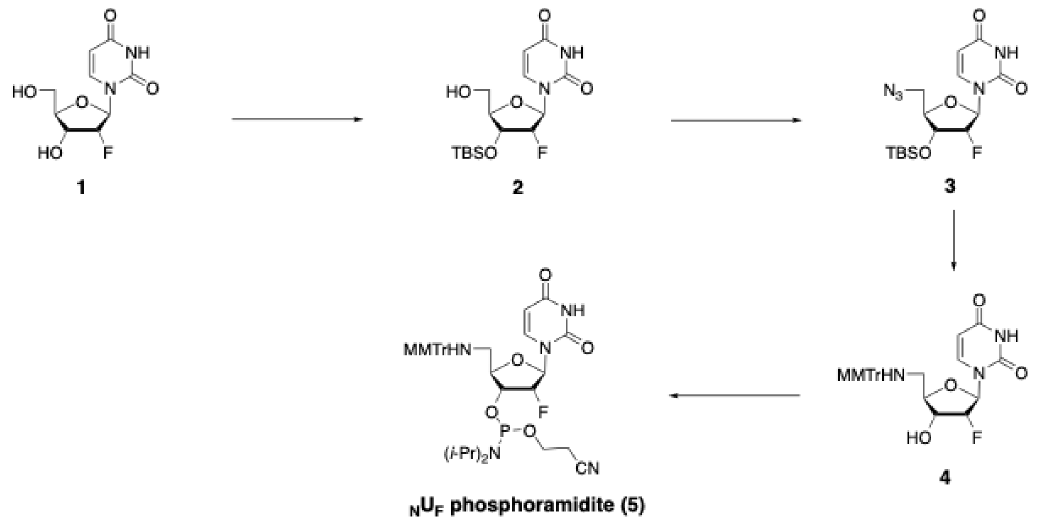

Figure S1. Synthetic scheme for ${}_{N}U_{F}$ phosphoramidite monomer.

Synthesis of ${}_{N}U_{F}$ phosphoramidite monomer (5)

2'-Fluoro-3'-*O*-(*tert*-butyldimethylsilyl)-2'-deoxyuridine (2)

A mixture of 2'-fluoro-2'-deoxyurideine (**1**) (1.23 g, 5.00 mmol), tert-butyldimethylchlorosilane (2.26 g, 15.0 mmol), and imidazole (2.04 g, 30.0 mmol) in *N,N'*-dimethylformamide (50 mL) was

stirred for 22 h at room temperature. To the mixture was added ethanol (5.0 mL) and the stirring was continued for another 30 min. The mixture was partitioned between AcOEt and H_2O, and the separated organic layer was washed three times with H_2O, and then with brine. After the organic layer had been dried with Na_2SO_4, it was concentrated in vacuo. The residue was then dissolved in a mixed solvent of THF-trifluoroacetic acid-H_2O (4:1:1, 60 mL) at 0°C, and stirred for 2.5 h at 0°C and then for 2 h at room temperature. To the mixture was added aqueous $NaHCO_3$ (saturated, 100 mL), and the whole was partitioned between AcOEt. The organic layer was washed with aqueous $NaHCO_3$ (saturated), H_2O, and brine. After the organic layer had been dried with Na_2SO_4, it was concentrated in vacuo, and the residue was purified with silica gel column chromatography (EtOH in $CHCl_3$) to give **2** (1.24 g, 69%) as a white solid; ESI-MS calcd. for $C_{15}H_{25}O_5N_2FSiNa$ $[M+Na]^+$: 383.14090, found: 383.14060; ^{1}H NMR (500 MHz, DMSO-d_6) δ: 11.40 (br s, 1 H), 7.89 (d, 1 H, J = 8.1 Hz), 5.90 (d, 1 H, J = 17.8 Hz), 5.64 (d, 1 H, J = 8.1 Hz), 5.23 (br s, 1 H), 5.11 (d, 1 H, J = 53.4 Hz), 4.33 (m, 1 H), 3.87 (m, 1 H), 3.76 (m, 1 H), 3.54 (m, 1 H), 0.88 (s, 9 H), 0.10 (s, 6 H,); ^{13}C NMR (125 MHz, DMSO-d_6) δ: 163.69, 150.79, 141.03, 102.15, 93.01 (J = 187.7 Hz), 87.92 (J = 34.4 Hz), 83.92, 69.19 (J = 15.5 Hz), 59.56 , 26.03 , 18.23, –4.45, –4.71.

5'-Azido-2'-fluoro-3'-*O*-(*tert*-butyldimethylsilyl)-2',5'-dideoxyuridine (3)

A mixture of 2'-fluoro-3'-*O*-(*tert*-butyldimethylsilyl)-2'-deoxyuridine (**2**) (1.24 g, 3.44 mmol) and *p*-toluenesulfonyl chloride (1.31 g, 6.88 mol) in pyridine (40 mL) was stirred for 3 days at room temperature. To the mixture was added H_2O (5.0 mL) and the stirring was continued for another 1 h. The mixture was concentrated in vacuo, and the residue was partitioned between AcOEt and H_2O. The separated organic layer was washed with aqueous $NaHCO_3$ (saturated), H_2O, and then with brine. After the organic layer had been dried with Na_2SO_4, it was concentrated in vacuo, and the residue was co-evaporated twice with toluene. The residue was then dissolved in *N,N'*-dimethylformamide (35 mL), sodium azide (610 mg, 9.39 mmol) and ammonium chloride (670 mg, 12.5 mmol) were added, and the mixture was stirred for 1 h at 80°C. The mixture was cooled to room temperature and was partitioned between AcOEt and H_2O. The separated organic layer was washed three times with H_2O, and then with brine. After the organic layer had been dried with Na_2SO_4, it was concentrated in vacuo, and the residue was purified with silica gel column chromatography (AcOEt in Hexane) to give **3** (1.03 g, 78%) as a white solid; ESI-MS calcd. for $C_{15}H_{25}O_4N_5FSiNa$ $[M+Na]^+$: 408.14738, found: 408.14709; ^{1}H NMR (500 MHz, DMSO-d_6) δ: 11.46 (br s, 1 H), 7.70 (d, 1 H, J = 8.0 Hz), 5.83 (d, 1 H, J = 22.4 Hz), 5.67 (d, 1 H, J = 8.0 Hz), 5.29 (dd, 1 H, J = 53.6, 3.8 Hz), 4.50 (m, 1 H), 3.95 (m, 1 H), 3.76 (d, 1 H, J = 13.8 Hz), 3.45 (dd, 1 H, J = 13.8, 4.6 Hz), 0.88 (s, 9 H), 0.12 (s, 3 H), 0.11 (s, 3 H); ^{13}C NMR (125 MHz, DMSO-d_6) δ: 163.68, 150.65, 142.87, 102.49, 92.48 (J = 186.1 Hz), 90.50 (J = 36.4 Hz), 81.02, 70.29 (J = 15.8 Hz), 50.48, 26.02, 18.20, -4.49, -4.75.

2'-Fluoro-5'-(4-monomethoxytritylamino)-2',5'-dideoxyuridine (4)

To a solution of 5'-azido-2'-fluoro-3'-*O*-(*tert*-butyldimethylsilyl)-2',5'-dideoxyuridine (**3**) (1.03 g, 2.67 mmol) in a mixed solvent of tetrahydrofuran-H_2O (9:1, 40 mL) was added triphenylphosphine (3 M in tetrahydrofuran, 1.78 mL, 5.34 mmol) and the mixture was stirred for 16 h at room temperature. To the mixture was added 1 N NaOH (2.7 mL) and the stirring was continued for another 1 h. The mixture was concentrated in vacuo, and the residue was partitioned between AcOEt and aqueous $NaHCO_3$ (saturated). The organic layer was then washed with brine. After the organic layer had been dried with Na_2SO_4, it was concentrated in vacuo. The residue was then dissolved in pyridine (35 mL), 4-monomethoxytrityl chloride (1.65 g, 5.34 mmol) was added, and the mixture was stirred for 18 h at room temperature. To the mixture was added aqueous EtOH (3.0 mL) and the stirring was continued for another 30 min. The mixture was concentrated in vacuo, and the residue was partitioned between AcOEt and H_2O. The separated organic layer was washed with aqueous $NaHCO_3$ (saturated) and brine. After the organic layer had been dried with Na_2SO_4, it was concentrated in vacuo, and the residue was co-evaporated twice with toluene. The residue was then dissolved in tetrahydrofuran (35 mL) at 0°C, tetrabutylammonium fluoride (1.0 M in tetrahydrofuran, 3.74 mL, 3.74 mmol) was added, and the mixture was stirred for 1 h at 0°C. To the mixture was added acetic acid (0.21 mL) and concentrated in vacuo. The residue was purified with silica gel column chromatography (EtOH in $CHCl_3$ containing 0.1% pyridine) to give **4** (996 mg, 72%) as a white form; ESI-MS calcd. for $C_{29}H_{28}O_5N_3FNa$ $[M+Na]^+$: 540.19052, found: 540.19032; ^{1}H NMR (500 MHz, DMSO-d_6) δ: 11.43 (br s, 1 H), 7.71 (d, 1 H, J = 8.1 Hz), 7.41 (d, 4 H, J = 7.7 Hz), 7.30-7.27 (m, 6 H), 7.18 (t, 2 H, J = 7.3 Hz), 6.85 (d, 2 H, J = 8.7 Hz), 5.85 (d, 1 H, J = 20.9 Hz), 5.57 (d, 1 H, J = 8.1 Hz), 5.51 (d, 1 H, J = 6.7 Hz), 5.11 (dd, 1 H, J = 53.5, 3.9 Hz), 4.18 (m, 1 H), 3.95 (m, 1 H), 3.72 (s, 3 H), 2.70 (t, 1 H, J = 8.0 Hz), 2.40 (m, 1 H), 2.27 (m, 1 H); ^{13}C NMR (125 MHz, DMSO-d_6) δ: 163.65, 157.89, 150.61, 146.63, 142.12, 138.16, 130.09, 129.37, 128.77, 128.68, 128.22, 126.56, 113.56, 102.14, 93.81 (J = 183.8 Hz), 89.57 (J = 35.6 Hz), 82.03, 70.18, 69.98 (J = 16.2 Hz), 55.44, 45.37.

3'-*O*-[(2-Cyanoethoxy)(*N*,*N*-diisopropylamino)phosphinyl]-2'-fluoro-5'-(4-monomethoxytritylamino)-2',5'-dideoxyuridine (5)

To a solution of 2'-fluoro-5'-(4-monomethoxytritylamino)-2',5'-dideoxyuridine (**4**) (500 mg, 0.97 mmol) in CH_2Cl_2 (20 mL) was added chloro(2-cyanoethoxy)(*N*,*N*-diisopropylamino)phosphine (0.54 mL, 1.94 mmol) and *N*,*N*-diisopropylethylamine (0.66 mL, 3.88 mmol), and the mixture was stirred for 50 min at room temperature. The mixture was partitioned between $CHCl_3$ and aqueous $NaHCO_3$ (saturated). The organic layer was then washed with H_2O and brine. After the organic layer had been dried with Na_2SO_4, it was concentrated in vacuo, and the residue was purified with silica gel column chromatography (AcOEt in Hexane containing 0.1% pyridine) to give **5** (520 mg, 75%) as a white form; ESI-MS calcd. for $C_{38}H_{45}O_6N_5FPNa$ $[M+Na]^+$: 740.29837, found: 740.29836; ^{31}P NMR (202

MHz, DMSO-d_6) δ: 149.45 (d, J = 6.9 Hz), 149.23 (d, J = 11.9 Hz).

2. Molecular weights of ligation products by phosphoramaidate-type ligation reaction.

Table S1

Name	calcd.	found	Δ
C_M-$_NU_{OH}$	7353.4423	7353.45	0.0077
C_M-$_NU_F$	7355.4337	7355.44	0.0063
C_F-$_NU_{OH}$	7341.4067	7341.42	0.0133
C_F-$_NU_F$	7343.3981	7343.42	0.0219
C_H-$_NU_{OH}$	7323.4163	7323.41	0.0063
C_H-$_NU_F$	7325.4077	7325.42	0.0123

3.Chemical ligation of RNA with a phosphate group at the 3' end (RNA3'P: C_X) and RNA with an amino group at the 5' end (5N'RNA: $_{N}U_{Y}$) on template DNA (dtm-A).

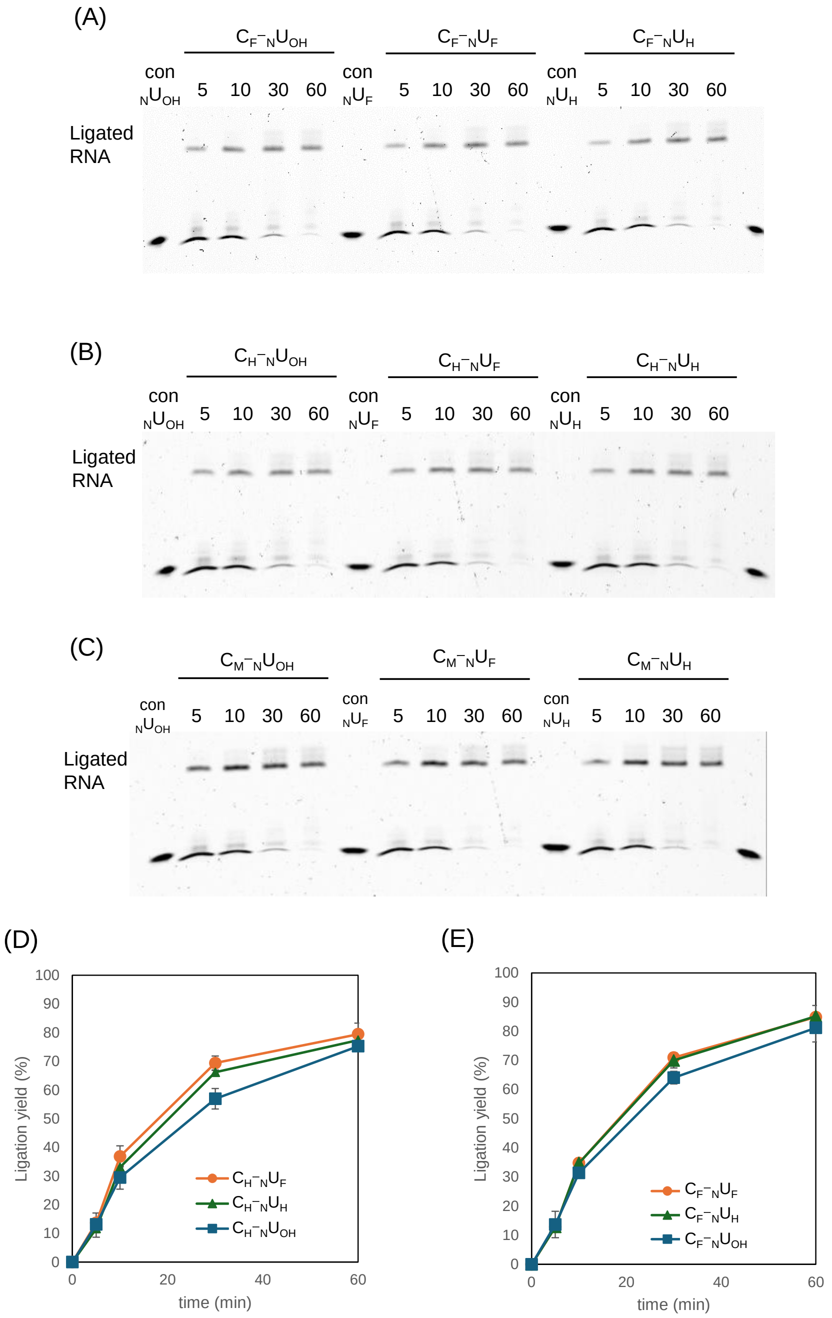

Figure S2. Chemical ligation of RNA with a phosphate group at the 3' end (RNA3'P: C_X) and RNA with an amino group at the 5' end (5N'RNA: $_{N}U_{Y}$) on template DNA (dtm-A). A solution containing 0.1 μM 3'P-RNA, 0.1 μM 5N'RNA and 0.1 μM dtm-A was incubated in 50 mM HEPES buffer (pH 7.2) containing 100 mM NaCl at 37 °C. PAGE analysis of ligation reaction of (A) C_F-$_{N}U_{Y}$, (B) C_H-$_{N}U_{Y}$, (C) C_M-$_{N}U_{Y}$. (D), (E) Time course of the ligation yield was determined by the fluorescence intensity of the product.

4.HPLC analysis of the ligation reaction between C_M and $_NU_F$.

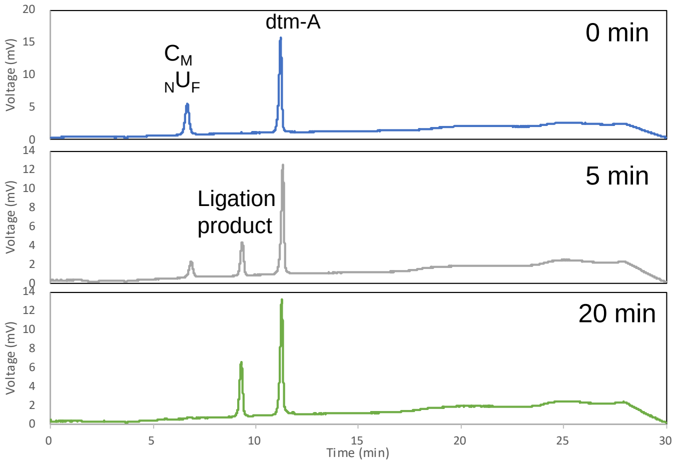

Figure.S3 HPLC analysis of the ligation reaction between C_M and $_NU_F$. A solution containing 1 μM C_M, 1 μM NUF and 1 μM dtm-A was incubated in 50 mM HEPES buffer (pH 7.2) containing 0.1 M NaCl at 37 ° C.

5.Chemical ligation of C_X and $_NU_Y$ on template RNA (rtm-A).

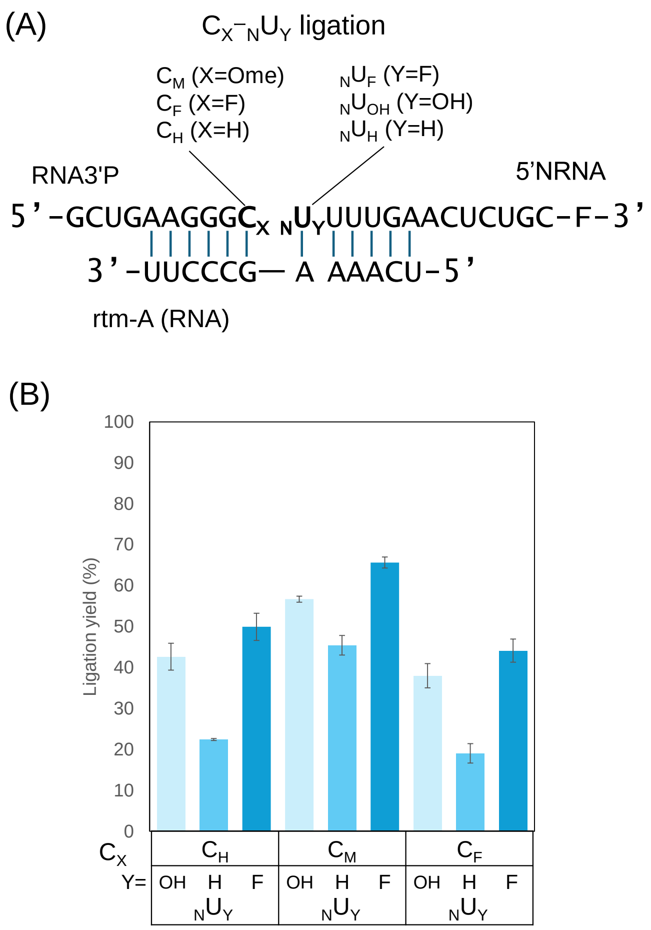

Figure S4. Chemical ligation of C_x and $_nU_Y$ on template RNA (rtm-A). A solution containing 1 μM RNA RNA3'P, 1 μM 5'NRNA and 1 μM rtm-A was incubated in 250 mM HEPES buffer (pH 7.2) containing 1 M NaCl at 27 °C. (A) Sequence of the strands and their 2'-modification of conjugation site. (B) Comparison of ligation ratio for 60 min reaction.

6. Chemical ligation of 3'-phosphorylated RNA (RNA3'P: C_X) and 5'-amino modified RNA (5'NRNA: ${}_NC_Y$) on template DNA (dtm-G).

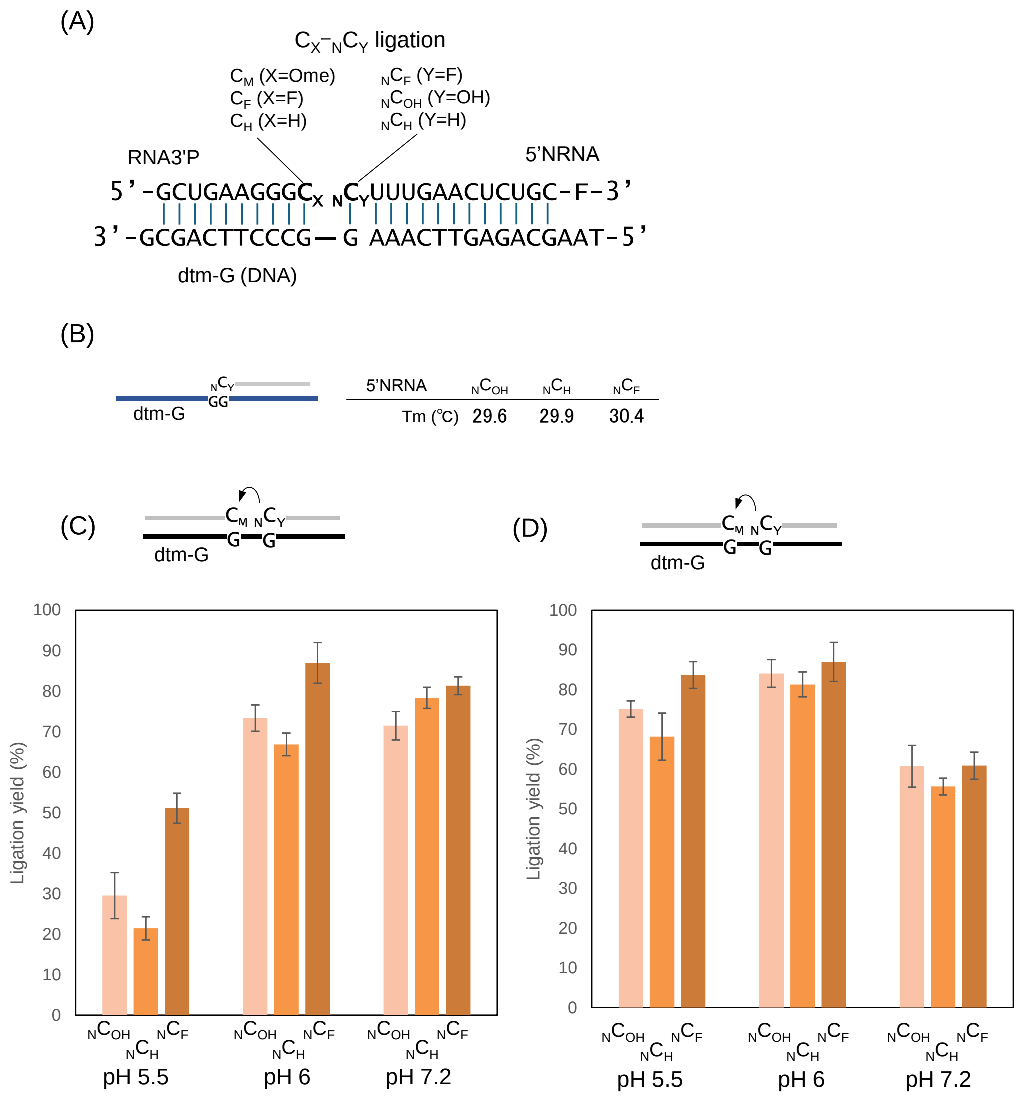

Figure S5. Chemical ligation of 3'-phosphorylated RNA (RNA3'P $=C_X$) and 5'-amino modified RNA (5'NRNA$={}_NC_Y$) on template DNA (dtm-G). (A) Sequence of the strands and their 2'-modification of conjugation site. (B) Tm values in hybridizing between dtm-G and 5'NRNA. Comparison of ligation ratio of C_M-${}_NC_Y$ for 30 min reaction at difference pH (C) at 37 ◦C and (D) 27 ◦C. A reaction solution containing 0.1 μM RNA3'P, 0.1 μM 5N'RNA and 0.1 μM template DNA was incubated in 50 mM MES (pH 5.5, 6) or HEPES buffer (pH 7.2) containing 100 mM NaCl.

7. HPLC analysis of the ligation reaction between miCM and 5'N-miUF.

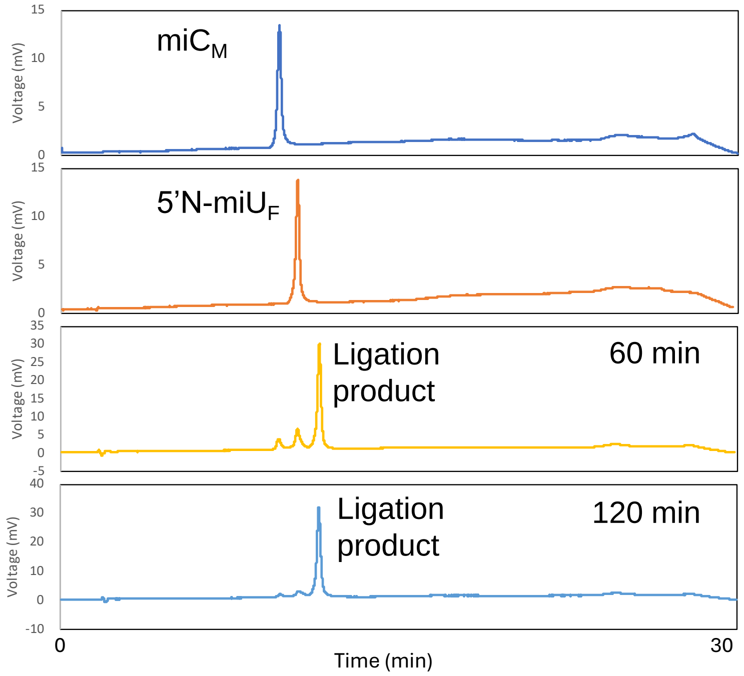

Figure.S6 HPLC analysis of the ligation reaction between miCM and 5’N-miUF. A solution containing 2 μM miCM, and 2 μM 5’N-miUF was incubated in 50 mM HEPES buffer (pH 7.2) containing 0.1 M NaCl at 27 ◦C.
